# Assessing the genetic overlap between BMI and cognitive function

**DOI:** 10.1038/mp.2015.205

**Published:** 2016-02-09

**Authors:** R E Marioni, J Yang, D Dykiert, R Mõttus, A Campbell, CHARGE Cognitive Working Group, Carla A Ibrahim-Verbaas, Jan Bressler, Stephanie Debette, Maaike Schuur, Albert V Smith, Gail Davies, David A Bennett, Ian J Deary, M Arfan Ikram, Lenore J Launer, Annette L Fitzpatrick, Sudha Seshadri, Cornelia M van Duijn, Thomas H Mosely Jr, G Davies, C Hayward, D J Porteous, P M Visscher, I J Deary

**Affiliations:** 1 Centre for Cognitive Ageing and Cognitive Epidemiology, University of Edinburgh, Edinburgh, UK; 2 Medical Genetics Section, Centre for Genomic and Experimental Medicine, Institute of Genetics and Molecular Medicine, University of Edinburgh, Edinburgh, UK; 3 Queensland Brain Institute, University of Queensland, Brisbane, QLD, Australia; 4 Department of Psychology, University of Edinburgh, Edinburgh, UK; 5 Generation Scotland, Centre for Genomic and Experimental Medicine, Institute of Genetics and Molecular Medicine, University of Edinburgh, Edinburgh, UK; 6 Medical Research Council Human Genetics Unit, Institute of Genetics and Molecular Medicine, University of Edinburgh, Edinburgh, UK; 7 University of Queensland Diamantina Institute, Translational Research Institute, University of Queensland, Brisbane, QLD, Australia

## Abstract

Obesity and low cognitive function are associated with multiple adverse health outcomes across the life course. They have a small phenotypic correlation (*r*=−0.11; high body mass index (BMI)−low cognitive function), but whether they have a shared genetic aetiology is unknown. We investigated the phenotypic and genetic correlations between the traits using data from 6815 unrelated, genotyped members of Generation Scotland, an ethnically homogeneous cohort from five sites across Scotland. Genetic correlations were estimated using the following: same-sample bivariate genome-wide complex trait analysis (GCTA)–GREML; independent samples bivariate GCTA–GREML using Generation Scotland for cognitive data and four other samples (*n*=20 806) for BMI; and bivariate LDSC analysis using the largest genome-wide association study (GWAS) summary data on cognitive function (*n*=48 462) and BMI (*n*=339 224) to date. The GWAS summary data were also used to create polygenic scores for the two traits, with within- and cross-trait prediction taking place in the independent Generation Scotland cohort. A large genetic correlation of −0.51 (s.e. 0.15) was observed using the same-sample GCTA–GREML approach compared with −0.10 (s.e. 0.08) from the independent-samples GCTA–GREML approach and −0.22 (s.e. 0.03) from the bivariate LDSC analysis. A genetic profile score using cognition-specific genetic variants accounts for 0.08% (*P*=0.020) of the variance in BMI and a genetic profile score using BMI-specific variants accounts for 0.42% (*P*=1.9 × 10^−7^) of the variance in cognitive function. Seven common genetic variants are significantly associated with both traits at *P*<5 × 10^−5^, which is significantly more than expected by chance (*P*=0.007). All these results suggest there are shared genetic contributions to BMI and cognitive function.

## Introduction

The obesity epidemic in the United Kingdom is a major public health problem. High body mass index (BMI), a marker of obesity, has been associated with an increased risk of multiple disease and health outcomes, such as type 2 diabetes and cardiovascular disease.^1, 2, 3^ It has also been associated with lower cognitive function.^4^ Possible mechanisms of this link include brain atrophy^5^ and type 2 diabetes,^6^ although the causality of such associations is not yet clear.^7^ Moreover, a recent study identified an association between increased BMI and a lower risk of dementia.^8^ Studies show genetic influences on both cognitive function^9^ and BMI.^10^ Twin models indicate inconsistent findings regarding the genetic correlation between the traits.^11, 12, 13^ Some report a genetic correlation of around 0.27 (such that genes for poorer cognitive performance correlate associate with genes for a higher BMI),^12^ others a genetic correlation of 0.12,^13^ whereas one found a null association.^11^ However, a genetic correlation has not yet been examined at the molecular genetic level. Identification of any shared genetic contributions could aid our understanding of the phenotypic association between lower cognitive function and higher BMI. This could also shed light on the aetiology of the health outcomes with which both are associated, such as increased mortality risk.^14, 15, 16, 17^

Molecular genetic studies have shown that common genetic variants explain around 30% of individual differences in cognitive function^9^ and around 10–20% of individual differences in BMI in adults (~30% in adolescents).^18, 19, 20^ However, this approach, using genome-wide complex trait analysis (GCTA–GREML), does not identify the specific variants and genes that contribute to the associations. One approach that uses information from specific genetic variants is polygenic scoring, which uses effect sizes (or, the strength of associations of different loci with the phenotype in question) from large genome-wide association studies (GWASs) to build linear predictors of the phenotype in independent cohorts. For example, previous studies have shown that a polygenic score for cognitive function (based on a GWAS of 48 462 people) predicts 1.27% of the variance in cognitive function in an independent cohort.^9^ One can also examine polygenic scores for correlated traits, for example, a higher polygenic score for schizophrenia is correlated with greater life-course cognitive decline.^21^

Here we examine the genotypic correlations between cognitive function and BMI. The genetic correlations are calculated using three different approaches: (1) bivariate GCTA–GREML^22, 23^ where both BMI and cognitive function are measured in the same sample; (2) bivariate GCTA–GREML wherein the traits are measured in different samples; and (3) LDSC regression,^24^ which uses summary GWAS data with potentially overlapping participants for each trait. We also relate polygenic risk scores for the two traits—predicting both within and across traits. Finally, we examine the overlap between existing GWAS analyses of both BMI and cognitive function, to identify individual single-nucleotide polymorphism (SNP) variants and genes that may be involved in shared biological pathways.

## Materials and methods

Data for the same-sample GCTA–GREML analysis, the phenotypic correlation analysis and the independent cohort for the polygenic prediction analysis came from Generation Scotland: the Scottish Family Health Study, a population-based, family-structured cohort that sampled over 24 000 people in Scotland between the years 2006 and 2011.^25, 26^ The study was set up for family-based genetic epidemiology research; health outcomes including coronary heart disease, stroke, cancer, chronic obstructive pulmonary disease, diabetes and mental illness are highly prevalent in Scotland. The sampling frame of the study focused on 7953 probands between ages 35–65 years, who were registered with participating general medical practitioners from five regional centres: Glasgow, Tayside, Ayrshire, Arran and the North-East of Scotland. The probands were invited to participate through the patient lists at the participating general medical practices; in the United Kingdom, ~96% of the population is registered with a general practitioner.^26^ Up to three generations of the probands' relatives were then recruited. There was no ascertainment bias towards a particular disease or health condition. A full description of the cohort has been given previously^25, 26^ and at www.generationscotland.org.

Cognitive function data for the independent-samples bivariate GCTA genetic correlation analysis came from Generation Scotland.^25, 26^ Open access data from dbGaP for the Gene Environment Association Studies initiative (GENEVA) project (comprising three studies, total *n*=14 347: Atherosclerosis Risk in the Community, Nurses' Health Study and the Health Professionals' Follow-up Study) and the Health and Retirement Study (*n*=8652) were used for the BMI analysis. Their dbGaP accession numbers are phs000090.v1.p1 (Atherosclerosis Risk in the Community), phs000091.v2.p1 (GENEVA-T2D) and phs000428.v1.p1 (Health and Retirement Study). A summary description of the three cohorts and details about quality controls of genotyped data and imputation can be found elsewhere.^27^

For the LDSC genetic correlation analysis, summary data from the largest GWAS studies to date for cognitive function^9^ and BMI^10^ were used.

### Generation Scotland ethical details

All components of Generation Scotland received ethical approval from the Nurses' Health Study Tayside Committee on Medical Research Ethics (REC Reference Number: 05/S1401/89). Generation Scotland: Scottish Family Health Study has also been granted Research Tissue Bank status by the Tayside Committee on Medical Research Ethics (REC Reference Number: 10/S1402/20), providing generic ethical approval for a wide range of uses within medical research.

### BMI in Generation Scotland

BMI was measured as weight in kilograms divided by height in metres squared (measurement details in Supplementary Information File S1). Participants with a BMI <17 or >50 were considered as outliers and were removed before the analyses.

### Cognitive function in Generation Scotland

A general cognitive factor was obtained via a principal component analysis of four cognitive tests that measured processing speed (Wechsler Digit Symbol Substitution Task^28^), verbal declarative memory (Wechsler Logical Memory Test; sum of immediate and delayed recall of one paragraph^29^), executive function (phonemic Verbal Fluency Test; using the letters C, F and L, each for 1 min^30^) and vocabulary (the Mill Hill Vocabulary Scale; junior and senior synonyms combined^31^). The first unrotated principal component, which explained 42% of the variance of the four tests, was extracted and used as the cognitive variable of interest. Three of the four cognitive tests that were used to derive the general cognitive factor were based on verbal stimuli (Verbal Fluency Test, Mill Hill Vocabulary Scale and the Logical Memory Test); however, they, along with the Digit Symbol test, targeted different domains of cognitive function: executive function, vocabulary, memory and processing speed, respectively. The statistically derived general cognitive factor therefore includes common variance from these four different facets of cognitive function.

### Generation Scotland genotyping

Genome-wide genotyping data were measured on a sub-sample of 10 000 participants using the Illumina HumanOmniExpressExome-8 v1.0 DNA Analysis BeadChip and Infinium chemistry.^32^ Measurement details and quality-control steps are reported in Supplementary Information File S1. After quality control, there was an analysis sample of 6815 unrelated individuals. SNPs with a minor allele frequency below 1% were excluded before the analysis, to prevent rare variants having an influence on the downstream analyses.

### Generation Scotland, GENEVA and Health and Retirement Study imputation and quality control

Genotype data in Generation Scotland, GENEVA and the health and retirement study were imputed to either HapMap2 or 1000G. Imputation details and quality control steps are reported in Supplementary Information File S1. After quality control, there were 27 791 unrelated individuals for analysis in the combined data set. Both phenotypes were adjusted for age in each gender group in each cohort separately. As the genotype data were imputed based on different reference panels, we included in the analysis only the SNPs in common with the HapMap3 panel, because the HapMap3 SNP set was optimised to capture common genetic variation in the human genome.^33^

### Statistical analyses

All phenotypic data analyses were conducted on the unrelated Generation Scotland cohort who had genome-wide genotyping data available (*n*=6815). To determine the associations between cognitive function and BMI, a linear model was used with general cognitive factor as the independent variable. Age and sex were included as covariates.

Age-, sex- and population stratification-adjusted residuals for general cognitive function and BMI were computed by linear regression. A conservative number (fourteen) of ancestry components were included.^34^ The residual values were carried forward to genome-wide complex trait analyses—GCTA-GREML^22, 35^—to obtain the proportion of variation in the variables explained by common SNPs. The univariate GCTA-GREML estimates for general cognitive function have been reported previously.^34^

Three methods were used to estimate the genetic correlation between BMI and general cognitive function. First, bivariate GCTA-GREML^23^ was run in Generation Scotland where the phenotypic and genotypic information came from the same unrelated individuals. This approach estimates the extent to which genetic similarities correlate with phenotypic similarities. However, the relatively small sample size (and corresponding large s.e.) for this analysis resulted in an imprecise estimate. Second, bivariate GCTA-GREML analysis^23^ was used on cognitive data from Generation Scotland and BMI data in American adults from four publicly available data sets. This approach estimates the genetic correlation through the SNP/phenotypic similarities in the independent samples. Third, summary GWAS output from the Davies *et al.*^9^ and Locke *et al.*^10^ papers were used to estimate the genetic correlation via the LDSC regression method.^24^ This method does not require raw genotype or phenotype information, and nor does it matter if there is an overlap of individuals in the two GWAS analyses. Briefly, this approach uses Linkage Disequilibrium (LD) structure (SNPs in regions of high LD will tag a greater part of the genome than those in low LD) whereby a SNP's association with a phenotype will result from its individual contribution and that of the surrounding SNPs in LD with it. In a bivariate setting, the expectation of the product of the statistical scores (*z*-scores) for the SNP–phenotype associations can be expressed as an intercept term and another term, including the genetic covariance between the traits, which does not depend on sample overlap for the input GWAS data.

A polygenic score for general cognitive function was calculated using data from a GWAS of general cognitive function (*n*=48 462);^9^ Generation Scotland did not contribute to the meta-GWAS. The greatest proportion of variance (1.27%, *P*=1.5 × 10^−17^) explained in general cognitive function was for a predictor that used SNPs with a *P*-value <0.5 in creating the score.^9^ Here we use the same predictor. For a brief summary of polygenic risk scoring, please see Supplementary Information File S2.

A polygenic score for BMI was created using summary data from a recent meta-analysis, which included 339 224 individuals.^10^ Generation Scotland was not included in the study. The greatest proportion of phenotypic variance in BMI is explained by a predictor that contains a subset of all HapMap 3 SNPs.^10^ We applied this predictor to our data.

Linear regression models were used to assess the relationship between the phenotypes and the polygenic scores, controlling for age, sex and population stratification (the first 14 prinicipal components (PCs)). The polygenic scores were pre-adjusted for age, sex and the 14 PCs with the residuals being used in the main models.

Using the results from the polygenic prediction analysis, we can provide estimates for the genetic correlation between cognitive function and BMI, based on their theoretical relationships (Supplementary Information File S3).

The GWAS output from the general cognitive function and BMI studies were merged to identify SNPs common to both analyses (Supplementary Information File S4). Significant hits at a suggestive threshold of *P*<5 × 10^−5^ in both studies were carried forward as potential polygenic variants that are important for individual differences in both traits. The total number of hits observed was compared with the expected number, based on an assumption of the two traits being independent.

Analyses were carried out in R.^36^ The polygenic risk scores were created using Plink.^37, 38^

## Results

A summary of the Generation Scotland cohort is presented in Table 1. The cohort had a median (interquartile range) age of 57 (49–63) years. Fifty-nine per cent of the cohort was female and the median education attained was 12–13 years. The mean BMI of the cohort was in the overweight range: 27.1 (s.d. 4.9) kg m^−2^. The summary data (means and s.d.) for the four cognitive tests that were used in the construction of the general cognitive factor are also presented in Table 1.

The age- and sex-adjusted linear regression model (Table 2) yielded a standardised effect size (*β*) of −0.10 (s.e. 0.01, *P*=1.3 × 10^−14^, *n*=6273) between the phenotypic measures of general cognitive function and BMI—better cognitive function is correlated with lower BMI. There was no evidence for a non-linear association between cognitive function and BMI after controlling for age and sex (*P*=0.090). A box plot showing the distribution of cognitive function scores by BMI decile is presented in Supplementary Information S5.

Estimates of the SNP-based heritabilities are presented in Table 3. The first approach, using data from the Generation Scotland sample alone, found univariate estimates, which represent the proportion of variance in the traits explained by common genetic variants, of 29% (s.e. 6%) for cognitive function and 28% (s.e. 6%) for BMI. The estimates for the second approach, which used data from Generation Scotland for cognitive function and the four US-based cohorts for BMI, were 31% (s.e. 5%) for cognitive function and 22% (s.e. 2%) for BMI. The estimates for the third approach (LD scoring), which used summary GWAS data from the Davies *et al.*^9^ and Locke *et al.*^10^ papers, were substantially lower for both traits: 15% (s.e. 1%) for cognitive function and 14% (s.e. 1%) for BMI.

Estimates of the genetic correlation between cognitive function and BMI for the three approaches are also reported in Table 3. The first method, bivariate GCTA-GREML using data from Generation Scotland for both traits, yielded a genetic correlation of −0.51 (s.e. 0.15). The estimate of the same genetic correlation was −0.10 (s.e. 0.08) using the independent-samples GCTA-GREML (Generation Scotland data for cognitive function, GENEVA and Health and Retirement Study data for BMI). The estimate for the third approach (LDSC regression), which used the summary GWAS data from Davies *et al.*^9^ and Locke *et al.*^10^ was −0.22 (s.e. 0.03). All three estimates consistently indicate that the genes associated with better cognitive function are also associated with a lower BMI.

The polygenic predictions, which were built using the GWAS summary data from the Davies *et al.*^9^ and Locke *et al.*^10^ GWASs and applied to the Generation Scotland cohort, are shown in Table 4. The polygenic score for general cognitive function predicted general cognitive function, explaining 0.81% of its variance (*P*=3.3 × 10^−13^, *n*=6273). The polygenic score for general cognitive function also predicted 0.08% of the variance in BMI (*P*=0.020, *n*=6463). The polygenic predictor for BMI explained 7.1% of the variance in BMI (*P*<2 × 10^−16^, *n*=6463), consistent with that reported previously,^10^ and 0.42% of the variance in general cognitive function (*P*=1.9 × 10^−7^, *n*=6273).

An analysis of the overlapping SNP variants from the cognition and BMI meta-GWASs identified seven variants (from fours genes: *AKAP6*, *TOMM40*, *TMEM161B* and *TNRC6B*) that were significant for both traits at *P*<5 × 10^−5^, which was greater than by chance (*P*=0.007; Supplementary Information File S4).

## Discussion

This study found an overlap of genetic influences on two important correlates of health outcomes over the life course: BMI and cognitive function. The phenotypic correlation between the traits was −0.11, indicating that better cognitive function is associated with lower BMI. The three estimates of the genetic correlation ranged between −0.51 and −0.10. A genetic correlation quantifies how genetic variants in one trait are correlated with genetic variants for another trait, averaged over the genome. Here, the gene variants associated with increased cognitive function scores were associated with lower BMI. We also showed, using polygenic risk score predictors derived from independent studies, that individual common genetic variants associated with BMI explain a significant proportion of the variance in cognitive function and vice versa. These proportions (0.42 and 0.08%) are very small. However, when they are compared with the proportions of variance that each polygenic risk score explained in their own respective phenotype (0.81 and 7.1%), this makes the former appear more substantial. There are seven individual genetic variants (four independent) that are associated with both traits at *P*<5x10^−5^, which is significantly more than expected by chance (*P*=0.007). Taken together, these findings point towards some shared biological underpinnings for BMI and general cognitive function.

The three empirical approaches taken to calculate the genetic correlation along with the theoretically derived estimate, based on the polygenic prediction results, is a strength of the study. Another strength is the novelty of the hypotheses being tested—using polygenic scores from BMI to predict general cognitive function and vice versa. Such analyses are important, as they aid our understanding of common sets of genetic variants that associate with multiple outcomes. We explored this further by examining the overlap of top hits from previous GWAS analyses of general cognitive function and BMI.

Although the Generation Scotland study had a large sample size, the same-sample genetic correlation still carried a relatively large s.e. Compared with the same-sample analysis, the independent-samples bivariate GCTA-GREML reduced the s.e. of the genetic correlation from 0.15 to 0.08, which then dropped further to 0.03 when we used the LDSC regression approach. With the decreasing s.e. came a convergence of the genetic correlation to an estimate of −0.22 from the LDSC regression analysis, which was contained in the 95% confidence intervals for the independent-sample and just inside of the same-sample GCTA-GREML interval. Again, it is worth noting that the same-sample GCTA-GREML estimate was measured with a lack of precision—its 95% confidence interval was (−0.80, −0.22). Theoretically, we would expect, given the polygenic prediction results, to have observed a genetic correlation of around −0.32 to −0.24 (Supplementary Information File S3), which is in line with the genetic correlation estimated from the LDSC regression analysis. One limitation of the Generation Scotland cohort for this study is the cross-sectional nature of the data. It may be the case that the association between BMI and cognitive function is diluted when looking in a cohort with a broad age range.

The univariate GCTA-GREML estimates obtained here are in accordance with those previously reported for general cognitive function^9^ and slightly higher for BMI.^18, 19^ The small within-trait polygenic prediction estimates correspond to those reported in the literature for cognitive abilities (~1%).^9^ The results accord with one of the predictions of the system integrity hypothesis, whereby cognitive function is hypothesised to be associated with health outcomes, because they all reflect a common general build quality of an organism.^39, 40, 41^ These results are also consistent with the finding that BMI-related diabetes is equally strongly associated with lower cognitive function before and after the onset of the disease.^7^ Larger meta-analysis GWAS studies for cognitive function and BMI will improve the predictive power of the polygenic predictors. Sequencing studies in very large samples that incorporate rare variants might also help us explain some of the missing heritability between molecular estimates of heritability and twin-based findings.

BMI and cognitive function are associated with numerous health outcomes.^1, 2, 3, 15, 42, 43^ Whereas the phenotypic correlation between the two traits is small, the genetic correlation is moderate, suggesting common biological pathways. Another possible explanation is that the associations reflect causal pathways. Techniques such as Mendelian Randomisation may help to tease apart determine the extent to which the pathways are shared versus linear (for example, genes to cognitive function, to BMI).^44^ The GWAS hits that are significant for both traits are found in genes linked to insulin-related processes (*AKAP6*), lipid transportation and Alzheimer's disease (*TOMM40*), retinal arterioral calibre (*TMEM161B*) and height (*TNRC6B*). The *TOMM40* SNP also tags the e4 allele defining SNP of *APOE*. Given the links between type 2 diabetes and impaired cognitive function, retinal microvascular disease and cognitive function, and height with cognitive function (and obviously BMI), these are plausible candidates that warrant further exploration. It is possible that there is an overlap in the anatomical substrate in the brain for the expression of the genes associated with both cognitive function and BMI.^10^ Future studies could consider downstream analyses to investigate whether these markers lie on causal pathways for the determination for either trait. For example, epigenetic marks such as DNA methylation have been identified as correlates of BMI in both blood and adipose tissue,^45^ as well as correlates of dementia in a case–control study of diabetics.^46^

Understanding the genetics of BMI and its overlap with the genetics of other correlates/predictors of health outcomes, for example, cognitive function, will help elucidate common pathways of disease outcomes. This study identified a small phenotypic correlation between BMI and cognitive function that is roughly half the size of the genetic correlation. Although genetic prediction of these traits is very small when applied to an individual, when coupled with the overlapping SNP hits for the traits they highlight shared genetic pathways for two important predictors of health outcomes, BMI and cognitive function.

## Figures and Tables

**Table 1:** Characteristics of the unrelated genotyped Generation Scotland cohort study members

		*Unrelated genotyped cohort*		
*Variable*	n	*Mean (s.d.) or* N *(%)*		*Range*
*Demographics*				
Age (years)	6463	57^a^	49–63	18–98
Sex: female	6463	3783	59	
Body mass index (kg m^−2^)	6463	27.1	4.9	17–50
				
*Cognitive function*				
Digit symbol test (0–133)	6379	68.5	16.7	0–133
Verbal fluency (0–inf)	6392	41.0	12.1	0–97
Logical memory (0–50)	6386	30.3	7.9	0–50
Mill Hill vocabulary scale (0–44)	6353	31.3	4.7	0–44

a Median (quartiles).

**Table 2:** General cognitive function associations with BMI

*General cognitive function*	n	*Beta*^a^	*s.e.*	P*-value*
Unadjusted association	6273	−0.11	0.01	<2.0 × 10^−16^
Adjusted for age and sex	6273	−0.10	0.01	1.3 × 10^−14^

Abbreviation: BMI, body mass index.

a The dependent variable and continuous independent variables were standardised in the regression models.

**Table 3:** Age-, sex- and population stratification-adjusted univariate and bivariate GCTA-derived and LDSC-derived estimates

*Univariate estimates*	n	*est*^a^	*s.e.*
*General cognitive function*			
Same-sample GCTA	6273	0.29	0.06
Independent-samples GCTA	6985	0.31	0.05
LDSC	48 462	0.15	0.01
			
*BMI*			
Same-sample GCTA	6463	0.28	0.06
Independent-samples GCTA	20 806	0.22	0.02
LDSC	339 224	0.14	0.01

*Bivariate estimates*	N	r_*G*_	*s.e.*
Same-sample GCTA	6273:6463	−0.51	0.15
Independent-samples GCTA	6985:20 806	−0.10	0.08
LDSC	48 462:339 224	−0.22	0.03

Abbreviations: BMI, body mass index; GCTA, genome-wide complex trait analysis; LDSC, Linkage Disequilibrium Score Regression; *r*_G_, genetic correlation.

a The proportion of variance in the phenotype explained by common genetic variants.

**Table 4:** Age- and sex-adjusted polygenic risk score associations with BMI and general cognitive function

	n	*Beta*^a^	*s.e.*	P*-value*
*General cognitive function polygenic score*^b^				
General cognitive function	6273	0.090	0.01	3.3 × 10^−13^
BMI	6463	−0.029	0.01	0.020
				
*BMI polygenic score*^b^				
General cognitive function	6273	−0.065	0.01	1.9 × 10^−7^
BMI	6463	0.266	0.01	<2 × 10^−16^

Abbreviation: BMI, body mass index.

a The dependent variable and continuous independent variables were standardised in the regression models.

b Polygenic risk scores were adjusted for age, sex and 14 multi-dimensional scaling components with residuals taken forward as the independent variable of interest.
