# Supplementary material for: Assessing the genetic overlap between BMI and cognitive function
Source: Mol Psychiatry. 2016 Feb 9;21(10):1477–82. doi: 10.1038/mp.2015.205 (PMC4863955; doi:10.1038/mp.2015.205)
Supplement: Supplementary Information [file mp2015205x1.doc]

**Supplementary Information**

Supplemental data includes five files with additional methods/results and an additional acknowledgements file.

**Supplementary Information 1 - Supplementary Methods**

**Supplementary Information 2 - Polygenic Scoring**

**Supplementary Information 3 - Across-trait correlation between phenotype and polygenic profile score.**

**Supplementary Information 4 - Overlapping SNP hits from the meta-GWAS's of cognitive function and BMI.**

**Supplementary Information 5 - Boxplot of cognitive function by BMI decile**

**Supplementary Information 6 - Additional acknowledgements**

**Supplementary Information 1 - Supplementary Methods**

*Body Mass Index in Generation Scotland*

Height was measured during clinical examination by asking each participant to remove their shoes and to stand (i) as erectly as possible with their back and shoulders against the freestanding measurement device, (ii) with heels together and feet angled at about 60 degrees, and (iii) with head held in the Frankfort horizontal plane, where the inferior border of the bony orbit is in line with the groove at the top of the tragus of the ear. Height to the nearest half centimetre was then measured during quiet breathing, with the horizontal arm of the measuring unit being kept at a rigid right angle to the scale. Weight was measured in kilograms; clothes weight was assumed to be 1kg, which was subtracted to give a finalized total.

*Generation Scotland Genotyping:*

Genome-wide data were collected on a sub-sample of 10,000 participants using the Illumina HumanOmniExpressExome-8 v1.0 DNA Analysis BeadChip and Infinium chemistry.^1^ Blood samples (or saliva from postal and a few clinical participants) from GS:SFHS participants were collected, processed and stored using standard operating procedures and managed through a laboratory information management system at the Wellcome Trust Clinical Research Facility Genetics Core, Edinburgh.^2^ The yield of DNA was measured using picogreen and normalised to 50ng/µl before genotyping. The Arrays were imaged on an Illumina HiScan platform and genotypes were called automatically using GenomeStudio Analysis software v2011.1. After quality control, there were a total of 594,824 single nucleotide polymorphisms (SNPs) available for analysis on 9,863 individuals, which included family trios and quads in addition to unrelated participants. A genetic threshold of 0.025 (between second and third cousins) was used to remove potential shared environment effects.^3,4^ This left an unrelated sample size of 6,815. SNPs with a MAF below 1% were excluded prior to the analysis.

*Generation Scotland, GENEVA, and Health and Retirement Study genotyping and QC*

In Generation Scotland, there were 9,140 individuals (including related subjects) with both genotype and cognitive phenotype data. The genotype data were imputed to HapMap2 using miniMACH. SNPs with Hardy-Weinberg Equilibrium (HWE) p < 1e-6, imputation quality (Rsq) < 0.6 or Minor Allele Frequency (MAF) < 0.01 were removed. The GENEVA genotype data had been imputed to 1000G. SNPs with HWE p < 1e-6, imputation Rsq < 0.6 or MAF < 0.01 were removed. Additional Quality Control was performed to remove SNPs with HWE p < 1e-6 in the combined GENEVA, Health and Retirement Study, and Generation Scotland data. After SNP QC, 971,719 SNPs were included in the analysis. We removed one of each pair of individuals with estimated genetic relatedness > 0.05, and retained 27,791 unrelated individuals for analysis in the combined data set. Both the BMI and cognitive phenotypes were adjusted for age in each gender group in each cohort separately. Since the genotype data were imputed based on different reference panels, we included in the analysis only the SNPs in common with the HapMap3 panel because the HapMap3 SNP set was optimized to capture common genetic variation in the human genome.^5^

**References**

1. Gunderson KL. Whole-genome genotyping on bead arrays. *Methods Mol. Biol.* 2009; **529**: 197-213.

2. Kerr SM, Campbell A, Murphy L, Hayward C, Jackson C, Wain LV et al. Pedigree and genotyping quality analyses of over 10,000 DNA samples from the Generation Scotland: Scottish Family Health Study. *BMC Med. Genet*, 2013; **14(1)**: 38.

3. Yang J, Benyamin B, McEvoy BP, Gordon S, Henders AK, Nyholt DR et al. Common SNPs explain a large proportion of the heritability for human height. *Nat. Genet.*, 2010; **42(7)**: 565-569.

4. Yang J, Lee SH, Goddard ME, Visscher PM. GCTA: a tool for genome-wide complex trait analysis. *Am. J. Hum. Genet.*, 2011; **88(1)**: 76-82.

5. The International HapMap3 Consortium. Integrating common and rare genetic variation in diverse human populations. *Nature*, 2010; **467**: 52-58.

**Supplementary Information 2 - Polygenic Scoring**

Polygenic scores utilise summary output from genome wide association studies to create prediction scores in independent cohorts. The GWAS output contains information on the genetic variants (the SNPs), the effect allele for the SNP, the regression weight per allele, and the P-value for the regression statistic. This can then be used in independent cohorts to create a score for each individual. For each SNP, the number of copies of the risk allele carried by the individual in the independent cohort will be multiplied by the regression weight from the initial GWAS. The scores are summed across SNPs for each individual to give the total risk score, a single number. This score can then be used as a predictor in subsequent analyses. In some circumstances, the best predictor of a trait will use information from only a subset of SNPs from the initial GWAS. This can be examined by using different thresholds (based on P-values e.g., <0.01, <0.05) for the SNPs.

**Supplementary Information 3 - Across-trait correlation between phenotype and polygenic profile score.**

This document outlines the calculations for the expected genetic correlation between BMI and cognitive function, given the observed cross-trait polygenic prediction results for the respective phenotypes.

*Assumptions and notation:*

y_i_ ~ N(0,1)

y_1_ = g_1_ + e_1_

y_2_ = g_2_ + e_2_

s_1_ = genetic predictor for trait 1 (from independent data)

s_2_ = genetic predictor for trait 2 (from independent data)

r_g_ = the correlation between g_1_ and g_2_, i.e. the genetic correlation

h_g1_ = sqrt(SNP heritability for trait 1), i.e. sqrt[var(g_1_) / var(y_1_)]

h_g2_ = sqrt(SNP heritability for trait 2) = sqrt [var(g_2_) / var(y_2_)]

R_1_ is the correlation between y_1_ and s_1_

R_2_ is the correlation between y_2_ and s_2_

R_12_ is the correlation between y_1_ and s_2_

*Derivation*

The accuracy of predicting y_1_ using s_2_ is R_12_ = cov(y_1_, s_2_) / sd(s_2_)

We know that

cov(y_1_, s_2_) = cov(g_1_, g_2_) = r_g_ * h_g1_ * h_g2_, assuming the SNPs are independent.

We also know from predicting trait 2 using genetic predictor for trait2 that

R_2_ = cov(y_2_, s_2_) / sd(s_2_)

Hence, sd(s_2_) = cov(g_2_, s_2_) / R_2_ = h_g2_^2^ / R_2_

Therefore,

R_12_ = (r_g_h_g1_)(R_2_/h_g2_)

The first term reflects how much genetic variation in trait 1 is captured by the SNPs and how the two traits are correlated, the second term reflect how accurate the genetic value for trait 2 that is captured by the SNPs is predicted.

If the traits are the same then r_g_ = 1 and h_g1_ = h_g2_ and R_12_ = R_2_. For a perfect predictor of trait 2 (R_2_ = h_g2_), R_12_ = h_g1_ = h_g2_.

Given observed R_12_ and R_2_, we can calculate

r_g_ = R_12_ * h_g2_ / (R_2_ * h_g1_)

For cognitive function (cog) and BMI, assuming h_g_^2^(cog) ~ 0.29 and h_g_^2^(BMI) ~ 0.28, this gives an expected value of the correlation between cognitive function and profile score (BMI), and vice versa, of,

R(y_BMI_, s_cog_) ~ r_g_ * R_cog_ and R(y_cog_, s_BMI_) ~ r_g_ * R_BMI_

If we assume R_cog_ = 0.09 and R_BMI_ = 0.27, then

R(y_BMI_, s_cog_) ~ 0.09 * r_g_ and and R(y_cog_, s_BMI_) ~ 0.27 * r_g_

The observed values of R(y_BMI_, s_cog_) are -0.029 and -0.065, respectively. These values are consistent with lower r_g_ values of -0.32 and -0.24, respectively.

If we assume r_g_ = -0.51 (but note that it is estimated with much error), then

R(y_BMI_, s_cog_) ~ -0.046 and R(y_cog_, s_BMI_) ~ -0.14, both of which are much larger than the observed values.

**Supplementary Information 4 - Overlapping SNP hits from the meta-GWAS's of cognitive function and BMI.**

In the Davies et al.^1^ cognitive function GWAS, SNP-cognition output associations were reported for 2,478,037 variants; the corresponding figure was 2,554,638 for the Locke et al.^2^ BMI GWAS. There was an overlap of 2,475,536 SNPs. Within this set, 812 were associated with general cognitive function and 6,649 were associated with BMI at P < 5x10^‑5^. Seven of the SNPs were common in both groups (rs17522122, rs2075650, rs2410767 - which was in high linkage disequilibrium (LD) with rs6870983 and rs7445169, and rs4820408 - in high LD with rs8142495). The expected number of hits common to both traits under an independence assumption was


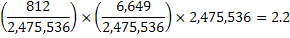


One thousand random draws of 812 and 6,649 SNPs from the 2,475,536 variants yielded an overlap of 7 or more SNPs on seven occasions, indicating a significant enrichment for hits in the empirical analysis (P=0.007). The seven SNPs identified were located in the following four genes: *AKAP6* (rs17522122), *TOMM40* (rs2075650), *TMEM161B* (rs2410767, rs6870983, rs7445169), and *TNRC6B* (rs4820408, rs8142495). The effect sizes for the SNPs in question were in opposite directions (variants that are positively associated with general cognitive function are negatively associated with BMI) with the exception of the *TOMM40* variant (Table 1). To investigate the *TOMM40* hit, which is in the same genomic region as *APOE* and tags the e4 defining SNP, rs429358, age- and sex-adjusted linear regression models were run for *APOE* status (e2e2, e2e3, e2e4, e3e3 (reference category), e3e4, e4e4) against BMI. All *APOE* haplotypes had lower BMI measures than the reference group (e3e3), although there was a significant difference in the e4e4 group alone (Table 2).

**References**

1. Davies G, Armstrong N, Bis JC, Bressler J, Chouraki V, Giddaluru S, *et al.* Genetic contributions to variation in general cognitive function: a meta-analysis of genome-wide association studies in the CHARGE consortium (N=53 949). Mol. Psychiatry 2015, Epub ahead of print.

2. Locke AE, Kahali B, Berndt SI, Justice AE, Pers TH, Day FR *et al.* Genetic studies of body mass index yield new insights for obesity biology. Nature 2015, **518(7538)**, 197-206.

Table 1: GWAS results from Locke et al. and Davies et al. for the seven SNPs that overlap at P<5x10^-5^

| **SNP** | **Gene** | **Effect allele** | **Beta_cognition_** | **P** | **Beta_BMI_** | **P** |
| --- | --- | --- | --- | --- | --- | --- |
| rs17522122 | *AKAP6* | T | -0.024 | 3.8x10^-7^ | -0.015 | 3.1x10^-6^ |
| rs2075650 | *TOMM40* | A | 0.055 | 8.4x10^-8^ | 0.026 | 1.2x10^-8^ |
| rs2410767 | *TMEM161B* | C | -0.033 | 2.0x10^-5^ | -0.018 | 3.5x10^-5^ |
| rs6870983 | *TMEM161B* | C | -0.033 | 2.6x10^-5^ | -0.018 | 4.2x10^-7^ |
| rs7445169 | *TMEM161B* | C | -0.033 | 2.2x10^-5^ | -0.018 | 4.3x10^-5^ |
| rs4820408 | *TNRC6B* | T | -0.025 | 2.3x10^-5^ | 0.015 | 3.6x10^-6^ |
| rs8142495 | *TNRC6B* | A | -0.023 | 2.6x10^-5^ | 0.014 | 6.8x10^-6^ |

Table 2: Age- and sex-adjusted associations between BMI and *APOE* haplotypes.

| ***APOE*** | **Beta** | **SE** | **P** |
| --- | --- | --- | --- |
| e2e2 | -1.51 | 0.83 | 0.07 |
| e2e3 | -0.26 | 0.20 | 0.18 |
| e2e4 | -0.10 | 0.41 | 0.80 |
| e3e3 | - | - | - |
| e3e4 | -0.20 | 0.15 | 0.19 |
| e4e4 | -0.84 | 0.40 | 0.04 |

BMI: body mass index

**Supplementary Information 5 - Boxplot of cognitive function by BMI decile.** Both BMI and cognitive function were adjusted for age, sex, and stratification (the first 14 principal components) prior to the analysis. The cognitive function residuals were then re-scaled to mean 0, standard deviation 1 for the plot.

**Supplementary Information 6 - Additional Acknowledgements**

**3C**

This work was made possible by the generous participation of the participants and their families. This work was supported by the National Foundation for Alzheimer’s disease and related disorders, the Institut Pasteur de Lille, the Centre National de Génotypage, Inserm, FRC (fondation pour la recherche sur le cerveau) and Rotary. This work has been developed and supported by the LABEX (laboratory of excellence program investment for the future) DISTALZ grant (Development of Innovative Strategies for a Transdisciplinary approachto ALZheimer’s disease). The Three-City Study was performed as part of a collaboration between the Institut National de la Santé et de la Recherche Médicale (Inserm), the Victor Segalen Bordeaux II University and Sanofi-Synthélabo. The Fondation pour la Recherche Médicale funded the preparation and initiation of the study. The 3C Study was also funded by the Caisse Nationale Maladie des Travailleurs Salariés, Direction Générale de la Santé, MGEN, Institut de la Longévité, Agence Française de Sécurité Sanitaire des Produits de Santé, the Aquitaine and Bourgogne Regional Councils, Fondation de France and the joint French Ministry of Research/INSERM “Cohortes et collections de données biologiques” programme. Lille Génopôle received an unconditional grant from Eisai.

**AGES**

The Age, Gene/Environment Susceptibility (AGES Reykjavik) Study was initiated to examine genetic susceptibility and gene/environment interaction as these contribute to phenotypes common in old age, and represents a continuation of the Reykjavik Study cohort begun in 1967. The Age, Gene/Environment Susceptibility Reykjavik Study has been funded by NIH contract N01-AG-12100, the NIA Intramural Research Program, Hjartavernd (the Icelandic Heart Association), and the Althingi (the Icelandic Parliament). The study is approved by the Icelandic National Bioethics Committee, (VSN: 00-063) and the Data Protection Authority. The researchers are indebted to the participants for their willingness to participate in the study.

**ARIC**

The Atherosclerosis Risk in Communities Study is carried out as a collaborative study supported by National Heart, Lung, and Blood Institute contracts (HHSN268201100005C, HHSN268201100006C, HHSN268201100007C, HHSN268201100008C, HHSN268201100009C, HHSN268201100010C, HHSN268201100011C, and HHSN268201100012C), R01HL087641, R01HL59367, and R01HL086694; National Human Genome Research Institute contract U01HG004402; and National Institutes of Health contract HHSN268200625226C. The authors thank the staff and participants of the ARIC study for their important contributions. Infrastructure was partly supported by Grant Number UL1RR025005, a component of the National Institutes of Health and NIH Roadmap for Medical Research.

**ASPS**

The authors thank the staff and the participants for their valuable contributions. We thank Birgit Reinhart for her long-term administrative commitment, Elfi Hofer for the technical assistance at creating the DNA bank, Ing. Johann Semmler and Anita Harb for DNA sequencing and DNA analyses by TaqMan assays and Irmgard Poelzl for supervising the quality management processes after ISO9001 at the biobanking and DNA analyses. The research reported in this article was funded by the Austrian Science Fund (FWF) grant number P20545-P05 and P13180. The Medical University of Graz supports the databank of the ASPS**.**

**BASE-II**

BASE-II has been financed by the Max Planck Society and the Federal Ministry of Education and Research. For a summary of the design of the study, see Bertram, L., Böckenhoff, A., Demuth, I., Düzel, S., Eckardt, R., Li, S.-C., Lindenberger, U., Pawelec, G., Siedler, T., Wagner, G. G., & Steinhagen- Thiessen, E. (2013). Cohort profile: The Berlin Aging Study II (BASE-II). Advance online publication. International Journal of Epidemiology.

**BETULA**

The Betula Study was supported by the Swedish Research Council to Lars-Göran Nilsson and Lars Nyberg (2001-6654, 2002-3794 and 2003-3883) and by a Wallenberg Scholar grant from the Knut and Alice Wallenberg Foundation to Lars Nyberg. Sudheer Giddaluru was supported by a grant from Helse Vest RHF to Stephanie Le Hellard (Grant 911554). We also thank the Centre for Advanced Study (CAS) at the Norwegian Academy of Science and Letters in Oslo for hosting collaborative projects and workshops between Norway, Sweden and Scotland in 2011-2012.

**CHS**

Cardiovascular Health Study: This CHS research was supported by NHLBI contracts HHSN268201200036C, HHSN268200800007C, N01HC55222, N01HC85079, N01HC85080, N01HC85081, N01HC85082, N01HC85083, N01HC85086; and NHLBI grants HL080295, HL087652, HL105756 with additional contribution from the National Institute of Neurological Disorders and Stroke (NINDS). Additional support was provided through AG023629 and R01AG15928 from the National Institute on Aging (NIA). A full list of principal CHS investigators and institutions can be found at CHS-NHLBI.org. The provision of genotyping data was supported in part by the National Center for Advancing Translational Sciences, CTSI grant UL1TR000124, and the National Institute of Diabetes and Digestive and Kidney Disease Diabetes Research Center (DRC) grant DK063491 to the Southern California Diabetes Endocrinology Research Center. The content is solely the responsibility of the authors and does not necessarily represent the official views of the National Institutes of Health.

**CROATIA Korcula and Split**

We would like to acknowledge the staff of several institutions in Croatia that supported the field work, including but not limited to The University of Split and Zagreb Medical Schools and the Croatian Institute for Public Health. We would also like to acknowledge the invaluable contributions of the recruitment teams in Korcula and Split, the administrative teams in Croatia and Edinburgh and the people of Korcula and Split.

**ERF**

The ERF study as a part of EUROSPAN (European Special Populations Research Network) was supported by European Commission FP6 STRP grant number 018947 (LSHG-CT-2006-01947) and also received funding from the European Community's Seventh Framework Programme (FP7/2007-2013)/grant agreement HEALTH-F4-2007-201413 by the European Commission under the programme "Quality of Life and Management of the Living Resources" of 5th Framework Programme (no. QLG2-CT-2002-01254). This study was financially supported by the Netherlands Organization for Scientific Research (NWO), the Internationale Stichting Alzheimer Onderzoek (ISAO), the Hersenstichting Nederland (HSN) and the Centre for Medical Systems Biology (CMSB) in the framework of the Netherlands Genomics Initiative (NGI) and by the Russian Foundation for Basic Research (RFBR). We thank the participants from the Genetic Research in Isolated Populations, Erasmus Rucphen Family, who made this work possible. Also, we thank Petra Veraart for collecting all genealogical data.

**FHS**

We acknowledge the National Heart, Lung, and Blood Institute, which has funded the SHARe (SNP Health Association Resource) project. A portion of FHS computations were using the Linux Cluster for Genetic Analysis (LinGA-II) funded by the Robert Dawson Evans Endowment of the Department of Medicine at Boston University School of Medicine and Boston Medical Center. We also acknowledge additional support from the NHLBI (Contracts No. N01-HC-25195; No. N02-HL-6-4278, R01HL93029, U01HL 096917), the National Institute of Aging (AG08122, AG16495; AG033193) and the NINDS (NS17950). The content is solely the responsibility of the authors and does not necessarily represent the official views of the National Institute on Aging, the National Institute of Neurological Disorders and Stroke, the National Heart Lung and Blood Institute or the National Institutes of Health.

**GENOA**

Support for the Genetic Epidemiology Network of Arteriopathy (GENOA) was provided by the National Heart, Lung and Blood Institute (HL054464, HL054457, HL054481, HL071917, and HL87660) and the National Institute of Neurological Disorders and Stroke (NS041558) of the National Institutes of Health. Genotyping was performed at the Mayo Clinic (S.T.T., Mariza de Andrade, Julie Cunningham) and was made possible by the University of Texas Health Sciences Center (Eric Boerwinkle, Megan L. Grove-Gaona). We would also like to thank the families that participated in the GENOA study.

**GS**

Generation Scotland has received core funding from the Chief Scientist Office of the Scottish Government Health Directorates CZD/16/6 and the Scottish Funding Council HR03006. We are grateful to all the families who took part, the general practitioners and the Scottish School of Primary Care for their help in recruiting them, and the whole Generation Scotland team, which includes interviewers, computer and laboratory technicians, clerical workers, research scientists, volunteers, managers, receptionists, healthcare assistants and nurses. Genotyping of the GS:SFHS samples was carried out by the Genetics Core Laboratory at the Wellcome Trust Clinical Research Facility, Edinburgh, Scotland and was funded by the UK’s Medical Research Council. REM and DJP undertook the work within the University of Edinburgh Centre for Cognitive Ageing and Cognitive Epidemiology, part of the cross council Lifelong Health and Wellbeing Initiative (MR/K026992/1). Funding from the BBSRC and Medical Research Council (MSRC) is gratefully acknowledged.

**HBCS**

We thank all study participants as well as everybody involved in the Helsinki Birth Cohort Study. Helsinki Birth Cohort Study has been supported by grants from the Academy of Finland, the Finnish Diabetes Research Society, Folkhälsan Research Foundation, Novo Nordisk Foundation, Finska Läkaresällskapet, Signe and Ane Gyllenberg Foundation, University of Helsinki, Ministry of Education, Ahokas Foundation, Emil Aaltonen Foundation.

**HCS**

The authors would like to thank the men and women participating in the HCS as well as The University of Newcastle, Vincent Fairfax Family Foundation and The Hunter Medical Research Institute.

**HRS**

HRS is supported by the National Institute on Aging (NIA U01AG009740). The genotyping was funded separately by the National Institute on Aging (RC2 AG036495, RC4 AG039029). Our genotyping was conducted by the NIH Center for Inherited Disease Research (CIDR) at Johns Hopkins University. Genotyping quality control and final preparation of the data were performed by the Genetics Coordinating Center at the University of Washington.

**LBC1921 and LBC1936**

We thank the cohort participants and team members who contributed to these studies. Phenotype collection in the Lothian Birth Cohort 1921 was supported by the UK Biotechnology and Biological Sciences Research Council (BBSRC), The Royal Society and The Chief Scientist Office of the Scottish Government. Phenotype collection in the Lothian Birth Cohort 1936 was supported by Research Into Ageing (continues as part of Age UK The Disconnected Mind project). Genotyping of the cohorts was funded by the BBSRC. The work was undertaken by The University of Edinburgh Centre for Cognitive Ageing and Cognitive Epidemiology, part of the cross council Lifelong Health and Wellbeing Initiative (MR/K026992/1). Funding from the BBSRC and Medical Research Council (MRC) is gratefully acknowledged.

**MAP and ROS**

The MAP and ROS data in the analysis is supported by National Institute on Aging grants P30AG10161, R01AG17917, R01AG15819, R01AG30146, the Illinois Department of Public Health, and the Translational Genomics Research Institute.

**NCNG**

The NCNG study has been funded through the Research Council of Norway (including the FUGE program), the National Institutes of Health, the University of Oslo, the University of Bergen, the Bergen Research Foundation (BFS), Helse Vest, and the Western Norway Regional Health Authority, the KG Jebsen Centre for Psychosis Research, and Dr. Einar Martens Fund. We also thank the Centre for Advanced Study (CAS) at the Norwegian Academy of Science and Letters in Oslo for hosting collaborative projects and workshops between Norway, Sweden and Scotland in 2011-2012.

**NHS & HPFS:** Funding support for the GWAS of Gene and Environment Initiatives in Type 2 Diabetes was provided through the NIH Genes, Environment and Health Initiative [GEI] (U01HG004399). The human subjects participating in the GWAS derive from The Nurses’ Health Study (NHS) and Health Professionals’ Follow-up Study (HPFS) and these studies are supported by National Institutes of Health grants CA87969, CA55075, and DK58845. Assistance with phenotype harmonization and genotype cleaning, as well as with general study coordination, was provided by the Gene Environment Association Studies, GENEVA Coordinating Center (U01 HG004446). Assistance with data cleaning was provided by the National Center for Biotechnology Information. Funding support for genotyping, which was performed at the Broad Institute of MIT and Harvard, was provided by the NIH GEI (U01HG004424).

**OATS**

We thank the OATS participants and gratefully acknowledge the support and assistance of the OATS Research Team. This work was facilitated by access to the Australian Twin Registry, a national research resource supported by the NHMRC Enabling Grant 310667 and administered by the University of Melbourne. DNA was extracted by Genetic Repositories Australia, an Enabling Facility, supported by the NHMRC Grant 401184. OATS genotyping was partly funded by a CSIRO Flagship Collaboration Fund Grant. Genome-wide genotyping was performed by the Diamantina Institute, University of Queensland. OATS is supported by the National Health and Medical Research Council of Australia (NHMRC) Project Grant 1045325 and the NHMRC/ARC Strategic Award 401162. KAM is supported by the NHMRC Capacity Building Grant 568940 and an Alzheimer’s Australia Dementia Research Foundation Postdoctoral Fellowship.

**ORCADES**

ORCADES was supported by the Chief Scientist Office of the Scottish Government, the Royal Society, the MRC Human Genetics Unit, Arthritis Research UK and the European Union framework program 6 EUROSPAN project (contract no. LSHG-CT-2006-018947). DNA extractions were performed at the Wellcome Trust Clinical Research Facility in Edinburgh. We would like to acknowledge the invaluable contributions of Lorraine Anderson and the research nurses in Orkney, the administrative team in Edinburgh and the people of Orkney.

**PROSPER**

The PROSPER study was supported by an investigator initiated grant obtained from Bristol-Myers Squibb. Prof. Dr. J. W. Jukema is an Established Clinical Investigator of the Netherlands Heart Foundation (grant 2001 D 032). Support for genotyping was provided by the seventh framework program of the European commission (grant 223004) and by the Netherlands Genomics Initiative (Netherlands Consortium for Healthy Aging grant 050-060-810).

**RSI, RSII and RSIII**

The generation and management of GWAS genotype data for the Rotterdam Study is supported by the Netherlands Organisation of Scientific Research NWO Investments (nr. 175.010.2005.011, 911-03-012). This study is funded by the Research Institute for Diseases in the Elderly (014-93-015; RIDE2), the Netherlands Genomics Initiative (NGI)/Netherlands Organisation for Scientific Research (NWO) project nr. 050-060-810. We thank Pascal Arp, Mila Jhamai, Marijn Verkerk, Lizbeth Herrera and Marjolein Peters for their help in creating the GWAS database, and Karol Estrada and Maksim V. Struchalin for their support in creation and analysis of imputed data. The Rotterdam Study is funded by Erasmus Medical Center and Erasmus University, Rotterdam, Netherlands Organization for the Health Research and Development (ZonMw), the Research Institute for Diseases in the Elderly (RIDE), the Ministry of Education, Culture and Science, the Ministry for Health, Welfare and Sports, the European Commission (DG XII), and the Municipality of Rotterdam. The authors are grateful to the study participants, the staff from the Rotterdam Study and the participating general practitioners and pharmacists.

**Sydney MAS**

We thank the Sydney MAS participants and the Sydney MAS Research Team for their support and assistance. DNA was extracted by Genetic Repositories Australia, an Enabling Facility, supported by the NHMRC Grant 401184. Genome-wide genotyping was performed by the Ramaciotti Centre, University of New South Wales. Sydney MAS is supported by the National Health and Medical Research Council of Australia (NHMRC) Program Grants 350833 and 568969. KAM, SR, NAK are supported by the NHMRC Capacity Building Grant 568940. NAK is also supported by a NHMRC Early Career Fellowship and KAM by an Alzheimer’s Australia Dementia Research Foundation Postdoctoral Fellowship.

**TASCOG**

National Health and Medical Research Council of Australia (NHMRC) Project Grants 403000, 491109; Heart Foundation/NHMRC Career Development Fellowship 606544 (VS); NHMRC Career development Fellowship (RS).

**GTEx Project**

The Genotype-Tissue Expression (GTEx) Project was supported by the Common Fund of the Office of the Director of the National Institutes of Health (commonfund.nih.gov/GTEx). Additional funds were provided by the NCI, NHGRI, NHLBI, NIDA, NIMH, and NINDS. Donors were enrolled at Biospecimen Source Sites funded by NCI\SAIC-Frederick, Inc. (SAIC-F) subcontracts to the National Disease Research Interchange (10XS170), Roswell Park Cancer Institute (10XS171), and Science Care, Inc. (X10S172). The Laboratory, Data Analysis, and Coordinating Center (LDACC) was funded through a contract (HHSN268201000029C) to the The Broad Institute, Inc. Biorepository operations were funded through an SAIC-F subcontract to Van Andel Institute (10ST1035). Additional data repository and project management were provided by SAIC-F (HHSN261200800001E). The Brain Bank was supported by a supplement to University of Miami grant DA006227. Statistical Methods development grants were made to the University of Geneva (MH090941), the University of Chicago (MH090951 & MH090937), the University of North Carolina - Chapel Hill (MH090936) and to Harvard University (MH090948). The datasets used for the analyses described in this manuscript were obtained from the GTEx Portal on 24 September 2014.

**International Genomics of Alzheimer's Project (IGAP)**

We thank the IGAP for providing summary results data for these analyses. The investigators within IGAP contributed to the design and implementation of IGAP and/or provided data but did not participate in analysis or writing of this report. IGAP was made possible by the generous participation of the control subjects, the patients, and their families. The i–Select chips was funded by the French National Foundation on Alzheimer's disease and related disorders. EADI was supported by the LABEX (laboratory of excellence program investment for the future) DISTALZ grant, Inserm, Institut Pasteur de Lille, Université de Lille 2 and the Lille University Hospital. GERAD was supported by the Medical Research Council (Grant n° 503480), Alzheimer's Research UK (Grant n° 503176), the Wellcome Trust (Grant n° 082604/2/07/Z) and German Federal Ministry of Education and Research (BMBF): Competence Network Dementia (CND) grant n° 01GI0102, 01GI0711, 01GI0420. CHARGE was partly supported by the NIH/NIA grant R01 AG033193 and the NIA AG081220 and AGES contract N01–AG–12100, the NHLBI grant R01 HL105756, the Icelandic Heart Association, and the Erasmus Medical Center and Erasmus University. ADGC was supported by the NIH/NIA grants: U01 AG032984, U24 AG021886, U01 AG016976, and the Alzheimer's Association grant ADGC–10–196728.
